# Two novel pathogenic *PDX1* variants in two Japanese patients with maturity-onset diabetes of the young

**DOI:** 10.1038/s41439-025-00312-4

**Published:** 2025-05-16

**Authors:** Satoshi Tanaka, Hiroyuki Akagawa, Michiyo Hase, Naoko Iwasaki

**Affiliations:** 1 Institute for Comprehensive Medical Sciences, Tokyo Women’s Medical University, Tokyo, Japan; 2 Diabetes and Metabolism, School of Medicine, Tokyo Women’s Medical University, Tokyo, Japan; 3 Department of Neurosurgery, Tokyo Women’s Medical University Adachi Medical Center, Tokyo, Japan; 4 Institute of Geriatrics, Tokyo Women’s Medical University, Tokyo, Japan; 5 Division of Diabetes, Endocrinology and Metabolism, Tokyo Women’s Medical University Yachiyo Medical Center, Chiba, Japan

**Keywords:** Diabetes, Disease genetics

## Abstract

Maturity-onset diabetes of the young type 4 (MODY4, PDX1-MODY) is a monogenic diabetes caused by the PDX1 gene. Here we detected two novel heterozygous missense variants, NM_000209.4(NP_000200.1):c.443G>T, p.(Arg148Leu) and c.442C>G p.(Arg148Gly), in two Japanese patients. Pathogenicity testing revealed a loss of function in both variants. Family members had severe diabetic complications, including proliferative retinopathy and overt nephropathy such as end-stage renal disease. Laboratory testing indicated persistently high glucose levels, at least partially caused by reduced postprandial insulin secretion.

## Introduction

Maturity-onset diabetes of the young (MODY) is a subtype of diabetes mellitus (DM) caused by a single gene disorder, mostly related to pancreatic β-cell development and function. The clinical characteristics of MODY include early onset of DM, typically before the age of 25 years, and no history of obesity or presence of autoantibodies against β cells despite the younger age of onset^1^. Advances in genomic research have enabled the identification of gene variants responsible for Mendelian diseases^2^. Pancreatic and duodenal homeobox 1 (*PDX1*) gene is the fourth causative gene of MODY, PDX1-MODY (MODY4). *PDX1* is a transactivator essential for pancreatic development and insulin secretion and is expressed mainly in the duodenum, gall bladder, pancreas, small intestine and stomach^3^. Information on the clinical characteristics and pathogenesis of the variants in patients with PDX1-MODY, however, is limited. Here, we report a large MODY family associated with a novel *PDX1* variant and a second family with the pathogenic variant previously reported but not analyzed on the basis of in vitro functional analysis.

## Case presentation

**Fig. 1:**
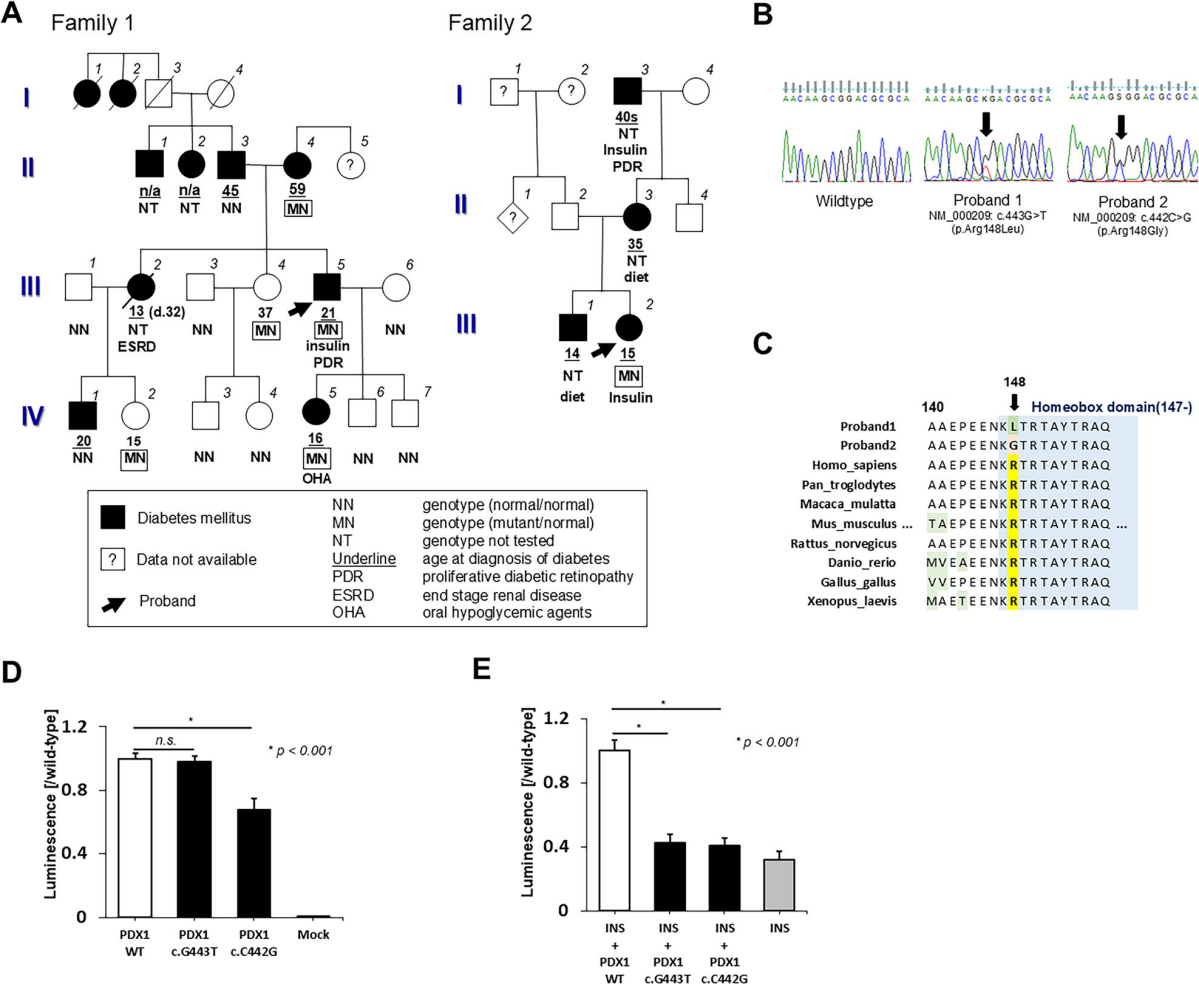
Pedigrees and variants information. **A** The pedigree of the probands. In family 1, the parents of the proband were diagnosed with DM—the father (II-3) at 45 years od age and the mother (II-4) at 59 years of age. The proband’s older sister (III-2) was diagnosed at 13 years of age and died at 32 years of age due to end-stage renal disease. His daughter (IV-5) developed DM at 16 years of age in 2010. Five family members, including the proband, were diagnosed with DM before 25 years of age. The grandfather (I-3) of proband 2 was diagnosed with DM in his 40 s and placed on insulin therapy. He had blindness due to diabetic retinopathy. **B** Sanger sequencing chromatograms. Top: the wild-type sequence as the control. Bottom: the variants (indicated by arrows) found in each proband. **C** The aspartic acid residue at this position is highly conserved across species. **D** A HiBiT luminescence assay showing intracellular PDX1 expression in HEK293T cells. Luminescence was significantly reduced in cells expressing PDX1:c.C442G p.(Arg148Gly) compared with those expressing wild-type PDX1 (*P* < 0.001). PDX1 wild type: 1.00 ± 0.03, PDX1 Arg148Leu: 0.99 ± 0.02, and PDX1 Arg148Gly: 0.68 ± 0.07. **E** Transcriptional activation of wild-type and mutant PDX1. Nluc luciferase activities were normalized against firefly luciferase activities used as the internal control. Insulin promoter and PDX1 wild type: 1.00 ± 0.05, insulin promoter and PDX1 Arg148Leu: 0.42 ± 0.04, insulin promoter and PDX1 Arg148Gly: 0.41 ± 0.06, and insulin promoter only: 0.31 ± 0.07.

Two Japanese probands were referred to our hospital for glycemic management. The pedigrees and clinical characteristics are presented in Fig. 1a and Table 1.

**Table 1:** Clinical characteristics of the probands.

	Proband 1	Proband 2
variant(NM_000209)	c.443G>T p.(Arg148Leu)	c.442C>G p.(Arg148Gly)
Age at onset (years), gender	21, male	15, female
Age at genetic test (years)	32	16
Height (cm)	168.8	161
Weight (kg)	61	53.6
BMI (kg/m^2^)	21.4	20.7
HbA1c (NGSP, %)	12.6	12.7
Plasma glucose (mg/dl)		
Fasting	180	192
Meal after 60 min	86	315
Meal after 120 min	157	418
Serum C-peptide (ng/ml)		
Fasting	1.8	1.2
Meal after 60 min	0.8	1.2
Meal after 120 min	1.9	1.7
Glucacon test		
Plasma glucose pre/6 min (mg/dl)	n/a	209/220
Serum C-peptide pre/6 min (ng/ml)	n/a	1.3/2.5
Urine C-peptide (μg/day)	32.1	24.7
GAD antibody	Negative	Negative
ICA antibody	Negative	Negative
Deep tendon reflex	Diminished	Diminished
Urinary albumin (mg/g Cr)	71.3	22.8
Retinopathy	Vitreous hemorrhage	No

BMI, body mass index; GAD, anti-glutamic acid decarboxylase; HbA1c, hemoglobin A1c; ICA, pancreatic islet cell; NGSP, National Glycohemoglobin Standarization Program; n/a, not available.

### Family 1

The proband was a 32-year-old man diagnosed with DM at the age of 27 years. Glycemic management was initially improved by diet therapy but deteriorated over time. While on glibenclamide (1.25 mg/day) and acarbose (300 mg/day), HbA1c levels ranged from 10.0% to 12.2%. At 31 years of age, he developed proliferative diabetic retinopathy and underwent vitrectomy.

### Family 2

The proband was a 16-year-old girl diagnosed with DM at 15 years of age and treated with gliclazide (80 mg/day), after which her HbA1c levels remained between 8.9% and 9.4%.

## Genetic and functional analyses

All the participants provided written informed consent. The Ethics Committee of Tokyo Women’s Medical University approved the study protocol. After obtaining genomic DNA, whole-exome sequencing was performed, and the outputs were processed in accordance with the Genome Analysis Toolkit (GATK) Best Practice Workflow (https://gatk.broadinstitute.org/, accessed 5 February 2025). We identified two novel heterozygous missense variants: NM_000209.4(NP_000200.1):c.443G>T (neither the rsID nor related information was available in ClinVar) in proband 1 and c.442C>G (rs193922355, https://www.ncbi.nlm.nih.gov/clinvar/variation/36407/, accessed 14 September 2024) in proband 2 (Fig. 1b and Supplementary Table 1). The aspartic acid residue at this position is highly conserved across species (Fig. 1c). In family 1, a segregation study was performed, revealing segregation in II-4, III-4, IV-2 and IV-5 (Supplementary Fig. 1). Notably, the proband’s daughter (IV-5) was diagnosed with DM at the age of 16 years, 7 years after genetic testing (Fig. 1a).

To evaluate the impact of the detected variants, we conducted transfection experiments. We compared intracellular *PDX1* expression using the HiBiT expression assay and found that the *PDX1* expression was lower in HEK293T cells expressing the c.442C>G variant than in those expressing wild-type *PDX1*. *PDX1* expression was comparable between cells expressing the c.443G>T variant and wild-type *PDX1*. The reporter assay demonstrated that wild-type *PDX1* increased the transcription of a luciferase reporter gene linked to the *PDX1* binding site of the human insulin promotor by 3.2-fold. By contrast, the *PDX1* variants (c.443G>T and c.442C>G) were nonfunctional, exhibiting activity comparable to that observed in HEK293T cells transfected with only a luciferase vector containing the insulin promoter. Detailed methods of the functional analyses of the detected variants are described in the Supplementary Information.

## Discussion

We identified two heterozygous variants, NM_000209.4(NP_000200.1):c.443G>T, p.(Arg148Leu) and c.442C>G p.(Arg148Gly), in the two Japanese probands with young-onset DM.

The c.443G>T variant in proband 1 has not been reported in any database, including the ClinVar database. The c.442C>G variant in proband 2 was reported as a ‘likely pathogenic’ variant (90% chance of pathogenicity) of PDX1-MODY in the ClinVar database (https://www.ncbi.nlm.nih.gov/clinvar/variation/36407/, accessed 14 September 2024). No additional information, including phenotype or function, has been reported. The reporter assay, a standard method for analyzing the function of transcription factors such as *PDX1*, showed a loss of function in p.Arg148Leu and p.Arg148Gly, both of which are considered loss-of-function variants. By contrast, in the expression assay using the HiBiT luciferase tag, intracellular expression of *PDX1* was reduced only in p.Arg148Gly and remained intact in p.Arg148Leu. One possible reason for these results is that the p.Arg148Leu variant affects mRNA and protein stability, leading to reduced intracellular *PDX1* expression. The Grantham matrix score^4^ is 102 for the change from Arg to Leu, and 125 for the change from Arg to Gly, suggesting that the Arg-to-Gly change has a greater impact on protein stability. This difference in stability could contribute to the observed results.

Based on the results of our functional study and the guidelines of the American College of Medical Genetics and Genomics (2015)^5^, the p.Arg148Leu variant in proband 1 fulfilled the criteria for ‘pathogenic’ using PS3+PM1+PM2+PP1+PP3. In proband 2, the pathogenicity of p.Arg148Gly has changed from ‘likely pathogenic’ to ‘pathogenic’ by fulfilling the criteria as PS3+PM1+PM2+PP3+PP5. Thus, both variants are considered pathogenic.

*PDX1* plays an essential role in the development and function of pancreatic β cells by transcriptionally regulating insulin expression. Heterozygous pathogenic variants of *PDX1* caused PDX1-MODY, and homozygous pathogenic variants lead to the development of neonatal DM caused by pancreatic agenesis^6^. Heterozygous *PDX1* variants could have a wide phenotypic distribution, including the age of onset and the frequency of diabetic complications. So far, more than 30 cases of PDX1-MODY or pancreatic agenesis have been reported in peer-reviewed journals; however, clinical information remains limited. In the Japanese population, only one case has been reported so far—a patient carrying the p.Leu73fs variant of *PDX1* (ref. ^7^), whose clinical features, particularly regarding postprandial insulin secretion impairment, are consistent with those of our cases. In family 1, the heterozygous variant c.443G>T p.(Arg148Leu) was co-segregated in most of the members exhibiting glucose intolerance; however, III-4 (37 years old) and IV-2 (15 years old) did not exhibit glucose intolerance at the time of the study. Nevertheless, future development of glucose intolerance cannot be ruled out. Yoshiji et al. reported that the degree of glucose intolerance in PDX1-MODY varies widely. In PDX1-MODY, some cases can be managed by diet alone or with oral hypoglycemic agents, while others present with severely reduced insulin secretion^7^, suggesting low penetrance of PDX1-MODY, although further investigation is needed. In addition, IV-1 was newly diagnosed with glucose intolerance in this study, although he did not share the variant. He was thus regarded as a phenocopy.

The phenotype of Arg148 amino acid variants is characterized by severe diabetic complications, such as proliferative retinopathy in proband 1 and the grandfather of proband 2 (I-3), and end-stage renal disease in sibling (III-2) of proband 1 (Fig. 1a). Both probands maintained basal insulin secretion; however, urine C-peptide levels were decreased. In proband 2, postprandial insulin secretion was also impaired, resulting in elevated postprandial blood glucose levels (Table 1). The severity of diabetic retinopathy was related to younger age at diagnosis^8,9^ and an increase in postprandial blood glucose^10^. Considering that *PDX1* variants can affect β-cell proliferation and survival^11^, in addition to increasing postprandial plasma glucose, the PDX1-MODY phenotype (at least the one caused by the Arg148 variant) might be similar to that of severe insulin-deficient DM^12^.

*PDX1* is a key homeodomain factor that regulates the transcription of insulin^13^, as well as somatostatin (*SST*), glucokinase (*GCK*), regulatory factor X6 (*RFX6*), hepatocyte nuclear factor 1-β (*HNF1B*) and *PDX1* (ref. ^14^), by forming a complex with transcriptional coactivators and contributing to glucose metabolism^15^. *PDX1* consists of a transactivation domain and a DNA-binding homeobox domain^16^. According to the ClinVar database and the current work, five of six (83%) pathogenic PDX1-MODY variants are located on the DNA-binding homeobox domain, while only one is located in the transactivation domain, indicating the importance of the homeobox domain in developing MODY. In addition, eight ‘likely pathogenic’ variants have been reported, of which 50% are located in the DNA-binding homeobox domain, including p.Arg148Gly (discussed in this study)^17,18^. The Arg148 residue comprises the N-terminal arm of the *PDX1* homeodomain and is important for DNA binding and specificity, as well as for stabilizing DNA binding by interacting with Arg148 and Arg188 (ref. ^19^). The pathogenicity of the p.Arg148 variant could be mediated through the disruption of DNA binding by altering the interaction between the N-terminal arm and phosphate backbone, leading to insulin deficiency. The effect of *PDX1* variants on molecular architecture warrants further investigations.

## HGV database

The relevant data from this Data Report are hosted at the Human Genome Variation Database at 10.6084/m9.figshare.hgv.3494 and 10.6084/m9.figshare.hgv.3497.

## Supplementary information

Supplementary Information
