# Supplementary material for: Two novel pathogenic PDX1 variants in two Japanese patients with maturity-onset diabetes of the young
Source: Hum Genome Var. 2025 May 16;12:8. doi: 10.1038/s41439-025-00312-4 (PMC12084518; doi:10.1038/s41439-025-00312-4)
Supplement: Supplementary file 1 — Supplementary Information [file 41439_2025_312_MOESM1_ESM.docx]

**SUPPORTING INFORMATION**

**Two novel pathogenic variants of PDX1 gene in two Japanese with maturity-onset diabetes of the young**

Satoshi Tanaka^1,2^, Hiroyuki Akagawa^1,3^, Michiyo Hase^4^, Naoko Iwasaki^1,2,4,5*^

^1^ Institute for Comprehensive Medical Sciences, Tokyo Women's Medical University, Tokyo, Japan

8-1, Kawada-cho, Shinjuku-ku, Tokyo, Japan. 162-8666

^2^ Diabetes and Metabolism, School of Medicine, Tokyo Women’s Medical University, Tokyo, Japan

8-1, Kawada-cho, Shinjuku-ku, Tokyo, Japan. 162-8666

^3^ Department of Neurosurgery, Tokyo Women's Medical University Adachi Medical Center, Tokyo, Japan

4-33-1, Ekita, Adachi-ku, Tokyo, Japan. 123-8558

^4^ Institute of Geriatrics, Tokyo Women's Medical University, Tokyo, Japan

2-15-1, Shibuya, Shibuya-ku, Tokyo, Japan, 150-0002

^5^ Division of Diabetes, Endocrinology and Metabolism, Tokyo Women's Medical University Yachiyo Medical Center, Chiba, Japan.

477-96 Owada Shinden, Yachiyo-shi, Chiba, Japan. 276-8524

*Corresponding author: Naoko Iwasaki, MD, PhD, FACP

[iwasaki.naoko@twmu.ac.jp](mailto:iwasaki.naoko@twmu.ac.jp)

## **Genetic analysis**

After obtaining informed consent, genomic DNA (gDNA) was extracted from peripheral blood leukocytes using a standard method.

In proband 1, exome capture was performed using SureSelect Human All Exon V5 Kit (Agilent Technologies Inc., Santa Clara, CA, USA) with a solution-based hybridization method. Enriched DNA libraries were sequenced with 100-bp paired-end reading on a HiSeq2000 sequencer (Illumina, San Diego, CA, USA). In proband 2, exome capture was performed using SureSelect Human All Exon V6 Kit and sequenced with 150-bp paired-end reading on a Novaseq6000 sequencer (Illumina).

The raw sequencing data of both ligands were aligned to the hg19 human genome build using Burrows-Wheeler Aligner (BWA)[e1]. Variants were identified and filtered following the best practices of the Genome Analysis Toolkit (GATK) and Variant Quality Score Recalibration (VQSR) [e2, e3]. Functional and allele frequency annotations were obtained using ANNOVAR[e4]. Additionally, Japanese-specific allele frequency data were obtained from the Human Genetic Variation Database (HGVD) [e5].

Candidate variants (**Table S1**) were validated through standard PCR-based amplification, followed by BigDye terminator cycle sequencing (Thermo Fisher Scientific, Waltham, MA, USA) on a 3130xl Genetic Analyzer (Thermo Fisher Scientific).

## **Segregation study**

For family 1, eight members underwent a 75-g oral glucose tolerance test (OGTT) to confirm glucose metabolism. Results of OGTT are shown in **Table S2**. Two siblings on the proband’s father’s side (Ⅱ-1 and Ⅱ-2) had diabetes (age at diagnosis unknown). At least two immediate family members were diagnosed with DM before 25 years of age. The proband was clinically diagnosed with MODY. Progressive complications of diabetes were apparent in several family members. Also, Sanger sequencing was performed for the remaining family members of proband 1 to confirm the segregation of PDX1:p.Arg148Leu. The primer sequences are listed in **Table S3**.

## **Quantitative analysis of cell expression (HiBiT assay)**

The effects of missense variants on the cell expression of *PDX1* were evaluated using the Nano-Glo® HiBiT Lytic Detection System (Promega Corporation, Madison, WI, USA). The *PDX1* transcript (NM_000209.4) sequence, which encodes pancreas/duodenum homeobox protein 1 (NP_000200), was obtained through PCR amplification using human duodenum cDNA from a multiple tissue cDNA panel (Takara Bio USA, Inc., San Jose, CA, USA) as a template and was then cloned into a pGEM-3Zf(+) Vector (Promega Corporation). The coding sequence CCDS9327.1 was subcloned into an SgfI/EcoICRI-digested pFC37K HiBiT CMV-neo Flexi® Vector (Promega Corporation). The missense variants in *PDX1* were introduced into the vector using KOD-Plus Mutagenesis Kit (TOYOBO Inc. Japan). Primer sequences used are listed in **Table S4**.

HEK293T cells were maintained in a humidified CO_2_ (5%) incubator at 37°C in high-glucose Dulbecco’s modified Eagle’s medium (DMEM) supplemented with 10% fetal bovine serum (FBS) and plated 24 h before transfection in 96-well plates. The cells were transfected with 200 ng of wild-type or mutant vector expressing *PDX1*, which was fused to the amino-terminal HiBiT tag, using Lipofectamine 3000 (Thermo Fisher Scientific Inc), according to the manufacturer’s protocol. The cells were incubated for 24 h, and the intracellular expression assay was performed according to the manufacturer’s instructions. Luminescence was measured using a Plate CHAMELEON V reader (Hidex, Turku, Finland) and data were compared between cells transfected with mutant or wild-type plasmid. Data are presented as mean ± *SD* of four wells per condition. One-way ANOVA and Tukey’s post hoc tests was used for statistical comparison. Statistical significance was set at p < 0.05.

## **Quantitative evaluation of the effect of *PDX1* variants on insulin promoter activity (luciferase reporter assay)**

To assess the impact of missense variants on *PDX1* signaling, the promoter sequences of the human insulin gene, which includes the A1 and A3/4 sequences (binding sites for *PDX1*), was placed upstream of deep-sea shrimp Oplophorus luciferase (NanoLuc®, Nluc). The sequence, including the promoter region of human insulin gene (−365 to +40 bp), was obtained through PCR amplification using healthy human gDNA, which produced EcoRV/HindIII overhangs at both ends and was subcloned into an EcoRV/HindIII-digested pNL1.1 Vector (Promega Corporation) [pNL1.1-*INS*]. The pGL4.53[luc2/PGK] vector, encoding firefly luciferase (Fluc) downstream of the human phosphoglycerate kinase (PGK) promoter, was used as a control (Promega Corporation). Primer sequences used are listed in **Table S4**.

HEK293T cells were cultured as described; transfected with 400 ng of pNL1.1-*INS* and 50 ng of pGL4.53 vector expressing firefly luciferase, with or without 200 ng of wild-type or mutant vector expressing *PDX1* using Lipofectamine 3000, and cultured for 24 h. The reporter assay was performed using the Nano-Glo Dual-Luciferase Reporter Assay System according to the manufacturer’s protocol. The luminescence of each solution was detected in the same manner as described above and luciferase activity was normalized by the ratio of firefly luciferase activity to NanoLuc luciferase activity. Data represent the mean ± *SD* of four wells per condition. One-way ANOVA and Tukey’s post hoc tests was used for statistical comparison. Statistical significance was set at p < 0.05.

## **Classification of variants according to the American College of Medical Genetics and Genomics (ACMG) guidelines**

The pathogenicity of the detected variants was evaluated using the ACMG guidelines 2015 [e6]. In this study, PS3 was applied to assess the results of the expression assay. For PP3 classification, three in silico tools were used to predict the functional impact of missense variants: Sorting Tolerant From Intolerant (SIFT) [e7], Polymorphism Phenotyping v2 (PolyPhen2, HumDiv model) [e8], and Rare Exome Variant Ensemble Learner (REVEL) [e9]. The scores generated by these tools were obtained from dbnsfp4.2a using ANNOVAR. The cutoff values for the deleterious effects of missense variants were retrieved from a recent study by the ClinGen Sequence Variant Interpretation Working Group [e10]: SIFT score ≤ 0.001, PolyPhen2 HumDiv score ≥ 0.978, and REVEL score ≥ 0.644. In this study, PP3 was applied for missense variants having at least three deleterious ratings among the three in silico predictions. PP5 was applied for variants registered as likely pathogenic in the ClinVar database (https://www.ncbi.nlm.nih.gov/clinvar).

## References

e1. Li H. and Durbin R. (2009) Fast and accurate short read alignment with Burrows-Wheeler Transform. Bioinformatics, 25:1754-60.rui

e2.<https://gatk.broadinstitute.org/hc/en-us/articles/360035531112--How-to-Filter-variants-either-with-VQSR-or-by-hard-filtering> <accessed 2025-02-05>

e3. Van der Auwera GA, Carneiro MO, Hartl C, Poplin R, Del Angel G, *et al.* (2013) From FastQ data to high confidence variant calls: the Genome Analysis Toolkit best practices pipeline. *Curr Protoc Bioinformatics* 43:11.10.1-11.10.33.

e4. Wang K, Li M, Hakonarson H. ANNOVAR: functional annotation of genetic variants from high-throughput sequencing data. Nucleic Acids Res. 2010;38(16):e164.

e5. Higasa K, Miyake N, Yoshimura J, Okamura K, Niihori T, *et al.* (2016) Human genetic variation database, a reference database of genetic variations in the Japanese population. *J Hum Genet* 61: 547-53.

e6. Richards S, Aziz N, Bale S, et al. Standards and guidelines for the interpretation of sequence variants: a joint consensus recommendation of the American College of Medical Genetics and Genomics and the Association for Molecular Pathology. *Genet Med*. 2015;17(5):405-424.

e7. Adzhubei IA, Schmidt S, Peshkin L, Ramensky VE, Gerasimova A, Bork P, et al. A method and server for predicting damaging missense mutations. Nat Methods. 2010;7(4):248-9.

e8. Ioannidis NM, Rothstein JH, Pejaver V, Middha S, McDonnell SK, Baheti S, et al. REVEL: An Ensemble Method for Predicting the Pathogenicity of Rare Missense Variants. Am J Hum Genet. 2016;99(4):877-85.

e9. Pejaver V, Byrne AB, Feng BJ, Pagel KA, Mooney SD, Karchin R, et al. Calibration of computational tools for missense variant pathogenicity classification and ClinGen recommendations for PP3/BP4 criteria. Am J Hum Genet. 2022;109(12):2163-77.

**Figure. S1. Sanger Sequencing Chromatograms of family members of proband.1.**


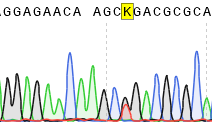


**II-4**

**
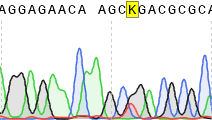
**

**III-4**

**
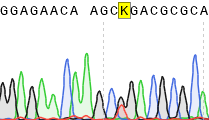
**

**III-5**

**
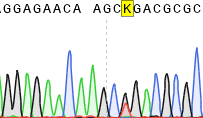
**

**IV-2**

**
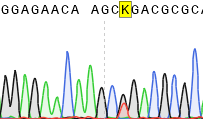
**

**IV-5**

| **Family** | **cytoBand** | **Coordinates (GRCh37)** | **Ref Allele** | **Variant Allele** |  | **Codon/Amino acid change (NM_000209.4)** | | **Variant Allele Frequency value** | | | **ACMG/AMP Criteria** | **SIFT score** | | **Polyphen2 score (HumDIV)** | | **REVEL score** |
| --- | --- | --- | --- | --- | --- | --- | --- | --- | --- | --- | --- | --- | --- | --- | --- | --- |
|  |  |  |  |  |  |  |  | **HGVD** | **gnomAD  PopFreqMax** | **1000 Genome  (East Asians)** |  |  |  |  |  |  |
| 1 | 13q12.2 | 28498429 | G | T |  | c.(443G>T) | p.(Arg148Leu) | 0 | 0 | 0 | PS3 + PM1 + PM2 + PP1 + PP3 | 0 | Deleterious | 1 | Damaging | 0.957 |
| 2 | 13q12.2 | 28498428 | C | G |  | c.(442C>G) | p.(Arg148Gly) | 0 | 0 | 0 | PS3 + PM1 + PM2 + PP3 + PP5 | 0 | Deleterious | 1 | Damaging | 0.911 |
| **Table S1: Variant information.** | | | |  |  |  |  |  |  |  |  |  |  |  |  |  |

| **No** | **Generation- Individual number** | **Gender** | **p.R148L variant** | **Age at 75g OGTT (years)** | **Age at diagnosis of DM (years)** | **Phenotype** | **HbA1c (%)** | **BMI (kg/m^2^)** | **PG in 75g OGTT  (mg/dL)** | | | | **Serum insulin levels  in 75g OGTT  (μIU/mL)** | | | |
| --- | --- | --- | --- | --- | --- | --- | --- | --- | --- | --- | --- | --- | --- | --- | --- | --- |
|  |  |  |  |  |  |  |  |  | **0 m** | **30 m** | **60 m** | **120 m** | **0 m** | **30 m** | **60 m** | **120 m** |
| 1 | II-3 | M | - | 65 | 45 | DM | 11.4 | 29.2 | 162 | - | - | - | 13.8 | - | - | - |
| 2 | II-4 | F | + | 62 | 59 | DM | 7.7 | 23.4 | 106 | - | - | - | 11.2 | - | - | - |
| 3 | III-1 | M | - | 42 | - | Normal | 5.7 | 25.3 | 93 | 152 | 113 | 79 | 17.4 | 24.1 | 38 | 29.8 |
| 4 | III-3 | M | - | 45 | - | Normal | 5.3 | 24.7 | 89 | 173 | 185 | 130 | 14.1 | 32.5 | 41 | 30.5 |
| 5 | III-4 | F | + | 37 | - | Normal | 5.6 | 27.1 | 98 | 119 | 97 | 116 | 13.4 | 21.3 | 19.9 | 19.7 |
| 6 | III-6 | F | - | 30 | - | Normal | 4.9 | n/a | 88 | 132 | 108 | 94 | 5.6 | 18.5 | 19.5 | 19.1 |
| 7 | IV-1 | M | - | 20 | 20 | DM | 11.8 | 30.4 | 212 | - | - | - | 23.5 | - | - | - |
| 8 | IV-2 | F | + | 15 | - | Normal | 5.3 | 21.9 | 88 | 132 | - | - | 22.1 | 32.5 | - | - |
| 9 | IV-3 | M | - | 16 | - | Normal | 4.9 | 23.9 | 76 | 129 | 94 | 82 | 11.9 | 46.7 | 46.7 | 18.5 |
| 10 | IV-4 | F | - | 15 | - | Normal | 4.7 | 25.6 | 73 | 113 | 102 | 87 | 11.6 | 21 | 20 | 23.4 |
| 11 | IV-5 | F | + | 9 | IGT at 9*, DM at 16 | IGT->DM | 5.2 at 9 11.9 at 16 | 20.1 at 16 | 85** | 199** | 164** | 133** | 16.9** | 106** | 89.2** | 87.1** |
| 12 | IV-6 | M | - | 6 | - | Normal | 5.2 | n/a | 91 | - | - | - | - | - | - | - |
| 13 | IV-7 | M | - | 3 | - | Normal | 5.3 | n/a | 78 | - | - | - | - | - | - | - |
|  | **Table S2: Results of the 75-g oral glucose tolerance test in family 1.** | | | | | |  |  |  |  |  |  |  |  |  |  |
|  | n/a: data not available, BMI: body mass index, PG: plasma glucose, OGTT: oral glucose tolerance test, DM: diabetes mellitus, IGT: impaired glucose tolerance | | | | | | | | | | | | | | | |
|  | *: IGT was diagnosed based on Japan Diabetes Society Criteria at that time | | | | | | |  |  |  |  |  |  |  |  |  |
|  | ** OGTT data of IV-5 at 9-year-old. | | | |  |  |  |  |  |  |  |  |  |  |  |  |

|  |  |  |  |
| --- | --- | --- | --- |
| Name | Sequence (5′->3′) | Target | Product size |
| PDX1_Ex2_F | CTACACTAGGCGCTGAAATG | c.G443T | 327 |
| PDX1_Ex2_R | TCTTGATGTGTCTCTCGGTC | c.C442G |  |
| **Table. S3**: Primers used for validating *PDX1* (NM_000209.4) variants. | | |  |

| Use | Primer title | Primer sequence (5′ -> 3′) |
| --- | --- | --- |
| *PDX1* cDNA cloning | PDX1_F2 | GGAACGCCACACAGTGCCAAA |
|  | PDX1_R2 | AACTGGCACATGCGCCTGAGA |
|  | EcoR1_PDX1_F2 | ATAGAATTCGGAACGCCACACAGTGCCAAA |
|  |  | *EcoRI* |
|  | Xba1_PDX1_R2 | TTATCTAGAACTGGCACATGCGCCTGAGA |
|  |  | *XbaI* |
| *INS [-365~+40bp]* cloning | newINSpromo_EcoRV | ATAGATATCGACAGCAGCGCAAAGAGC |
|  |  | *EcoRV* |
|  | newINSpromo_HindIII | TATAAGCTTGCTTGATGGCCTCTTCTGA |
|  |  | *HindIII* |
| pFC37K HiBiT insertion | PDX1F_Sgf1 | ATAGCGATCGCCATGCAATTGCCCCGGTGGT |
|  |  | *SgfI* |
|  | PDX1R_Pme1 | TTGGTTTAAACTAAATTCATTGCTTCAGTCAG |
|  |  | *PmeI* |
| Mutagenesis (c.G443T) | PDX1_R148L_5-3new | AGCTGACGCGCACGGCCTACA |
|  | PDX1_R148L_3-5new | TGTTCTCCTCCGGCTCCGCAG |
| Mutagenesis (c.C442G) | PDX1_R148G_5-3 | AGGGGACGCGCACGGCCTACA |
|  | PDX1_R148G_3-5 | TGTTCTCCTCCGGCTCCGCAGCG |
| **Table. S4:** Primers used for the reporter assay of c.G443T (p.Arg148Leu) and c.C442G (p.Arg148Gly) variants in *PDX1*. | | |
